# Answer to "Current Potential for Clinical Optimization of Social Cognition Assessment for Frontotemporal Dementia and Primary Psychiatric Disorders"

**DOI:** 10.1007/s11065-022-09556-1

**Published:** 2022-09-07

**Authors:** Alessandra Dodich, Chiara Cerami

**Affiliations:** 1 Center for Mind/Brain Sciences-CIMeC, University of Trento, 38068 Rovereto, TN Italy; 2 IUSS Cognitive Neuroscience ICoN Center, Scuola Universitaria Superiore IUSS Pavia, 27100 Pavia, Italy; 3 Cognitive Computational Neuroscience Research Unit, IRCCS Mondino Foundation, 27100 Pavia, Italy

The last version of the Diagnostic and Statistical Manual for Mental Disorders, that is, DSM 5th edition (DSM-5), a common framework for the clinical diagnosis of neurodevelopmental, neuropsychiatric, and neurodegenerative conditions reflecting specific underlying psychobiological dysfunctions, includes social cognition among the neurocognitive domains that can be impaired in neurocognitive disorders (NCDs). Socially inappropriate behaviors and other changes in social cognition subdomains have been proved in major (dementia) and minor (mild cognitive impairment) NCDs (see for examples Bora et al., 2015; Bora & Yener, 2017), supporting the impairment of social cognitive abilities even in a pre-dementia phase. In view of the increasing interest in social cognition as a possible cognitive marker of NCD syndromes, we recently reviewed the clinical validity of social cognition tasks in the diagnosis of the behavioral variant of frontotemporal dementia (bvFTD) (Dodich et al., 2021) a NCD syndrome in which social cognition deficits have been largely investigated and represent the core symptomatology (Rascovsky et al., 2011; Schroeter et al. 2014). Although a large body of literature actually supports the use of some neuropsychological tasks for the assessment of social cognitive subdomains in bvFTD, our systematic review notably showed that only a few patient-administered tools, for which accuracy measures (e.g., sensitivity, specificity) have been tested, are available in clinical practice (Dodich et al., 2021).

In their commentary "Current potential for clinical optimization of social cognition assessment for frontotemporal dementia and primary psychiatric disorders" Van den Stock and colleagues discuss the methodology and results of our systematic review. The authors place the right emphasis on the discrepancy between the large body of research studies available on this topic and the limited number of papers retained by the systematic review search. A Scopus search identified 2600 papers using frontotemporal dementia and social cognition as keywords (see Fig. 1 of Van den Stock et al., 2022), in comparison to 663 papers using a combination of FTD, social cognition and accuracy keywords, among which only 14 met the inclusion criteria (Dodich et al., 2021). Indeed, what is shown by the systematic review and underlined by Van den Stock and collaborators is the important mismatch between the flourishing results from experimental social cognition research in FTD and the poor implementation of guidelines and methodology in clinical setting. The underlying reasons for this slow translation of social cognitive markers into clinical practice could be manifold. Synergies between experimental social cognition research and clinical neuropsychology in dementia have been poor for decades, and research initiatives addressing cognitive markers development, standardization and multicultural validation in real-life clinical scenarios are still lacking. Overall, validation of diagnostic tests in dementia is at present still unsatisfactory, and professionals working in memory clinics should be fully aware of the clinical and ethical implications of the use of a diagnostic test with insufficient clinical validity (Frisoni et al., 2017). No consensus on the best tool to assess socio-cognitive deficits in NCDs and no clear guidelines for implementing the assessment of social cognition in memory clinics for the early or differential diagnosis are currently available. As a matter of fact, the use of validated socio-cognitive tasks is mostly limited to academic memory clinics with a specific interest in the field, while a relevant lack of knowledge is often observed in other clinical applications (e.g., Quesque et al., 2022; Jarsch et al., 2022), requiring a prompt response in terms of changes in practice and dissemination.

Giving the push for an increasingly early diagnosis (Barker et al., 2022), in our systematic review, we specifically focused on objective patient-administered social cognitive tasks. Although informant-based measures proved some utility in the diagnostic framework and in disease monitoring (Toller et al., 2020), and represent a useful proxy of social cognitive facets for which objective measures are not available (e.g., affective empathy), objective patient-administered social cognitive tasks have the advantage to provide information in subjects with no family informant. This might be of particular relevance in NCD patients in the very early disease stages and in at-risk subjects (e.g. carriers of known causative mutations) who usually refer alone to the specialist and for whom caregiver support is not required. However, the choice to use patient-administered or informant-based tools for the early or the differential clinical diagnosis of bvFTD (and of other NCDs) should be better investigated in multicultural comparative studies and then validated in different clinical settings, taking also into account possible cultural biases. In this sense, the multi-centric and multicultural initiative “Social and Affective Cognition workgroup within the Neuropsychiatric International Consortium for Frontotemporal Dementia (NIC-FTD)” introduced by Van den Stock and colleagues could include the optimal set of actions to obtain relevant advances in the field of FTD (Van den Stock et al., 2022).

The use of cognitive markers in dementia has radically changed in the last decade, in parallel with the translation from a clinical-neuropsychological to a biological diagnosis. Neuropsychological tests cannot be regarded as biological markers of pathology (Frisoni et al., 2017), but they can be a useful gatekeeper for better use of invasive or expansive biomarkers, with the final aim of reaching sufficient in vivo confidence in the diagnosis of NCDs reflecting an underlying psychobiological dysfunction. In view of these limitations, researchers may rather focus on testing clinical diagnostic use of social cognition measures, especially in milder patients, by supporting the detection of initial cognitive alteration with sufficient level of accuracy. As stated by Van den Stock et al., although relevant for clinical purposes, reports of sensitivity and specificity are often out of the aims of experimental social cognition studies (Van den Stock et al., 2021). The use of tests with poor sensitivity or specificity may finally result in a late diagnosis and delayed access to clinical trials, or in overdiagnosis and inappropriate overtreatment. A recent conjunct effort involving European researchers and clinicians (Boccardi et al., 2021) reached a consensus on a common standard neuropsychological assessment to detect mild NCD due to different etiologies in memory clinics, that is, the clinician’s Uniform Dataset (cUDS). Notably, the cUDS covers all the cognitive domains recommended by the DSM-5, including social cognition (Boccardi et al., 2021). This represented a revolution of perspective which fueled the debate on the assessment of social cognitive skills in NCD (Van den Stock et al., 2021; Dodich et al., 2022). On this constructive debate, within the cUDS consortium, the Neuropsychology working group, including experts in social cognition, moved forward laying the foundations of an inclusive multi-centric international initiative on the “clinical use of SocIal coGNnition measures for the AssessmenT of neURocognitivE disorders” (SIGNATURE initiative; https://sites.google.com/unitn.it/signature-initiative/home). The SIGNATURE initiative is aimed at promoting a constructive dialogue among methodology experts, social cognition researchers, memory clinic professionals (physicians and psychologists) and stakeholders (caregivers and patients), with the idea to boost a feasible and shared implementation of socio-cognitive tests in the clinical assessment of mild NCDs. The SIGNATURE roadmap embraces different phases, from the definition of clinical needs, up to the recommendations on clinical research priorities involving bottom-up users (clinicians and stakeholders) and top-down developers (experts). The consortium will definitely benefit from a combined top-down (experimental neuropsychology) and bottom-up (clinical neuropsychology) approach based on different memory clinic settings. The initiative is fully open and we invite any interested reader who would like to get involved to contact the consortium at signature.initiative@gmail.com. We believe that such initiative could be a valuable and unique opportunity to overcome current limitations and building more robust translational data for the ultimate benefit of NCD patients and caregivers.

## Data Availability

Not applicable.
